# Active ginseng components in cognitive impairment: Therapeutic potential and prospects for delivery and clinical study

**DOI:** 10.18632/oncotarget.26035

**Published:** 2018-09-11

**Authors:** Md. Jakaria, Md. Ezazul Haque, Joonsoo Kim, Duk-Yeon Cho, In-Su Kim, Dong-Kug Choi

**Affiliations:** ^1^ Department of Applied Life Science, Graduate School, Konkuk University, Chungju 27478, Republic of Korea; ^2^ Department of Integrated Bioscience & Biotechnology, College of Biomedical and Health Science, and Research Institute of Inflammatory Disease, Konkuk University, Chungju 27478, Republic of Korea

**Keywords:** cognitive impairment, ginseng, active components, potential

## Abstract

Cognitive impairment is a state that affects thinking, communication, understanding, and memory, and is very common in various neurological disorders. Among many factors, age-related cognitive decline is an important area in mental health research. Research to find therapeutic medications or supplements to treat cognitive deficits and maintain cognitive health has been ongoing. Ginseng and its active components may have played a role in treating chronic disorders. Numerous preclinical studies have confirmed that ginseng and its active components such as ginsenosides, gintonin, and compound K are pharmacologically efficacious in different models of and are linked to cognitive impairment. Among their several roles, they act as an anti-neuroinflammatory and help fight against oxidative stress and modulate the cholinergic signal. These roles may be involved in enhancing cognition and attenuating impairment. There have been some clinical studies on the activity of ginseng in cognitive impairment, but many ginseng species and active compounds remain to be investigated. In addition, new formulations of active ginseng components such as nanoparticles and liposomes could be used for preclinical and clinical models of cognitive impairment. Here, we discuss the therapeutic potential of active ginseng components in cognitive impairment and their chemistry and pharmacokinetics and consider prospects for their delivery and clinical study with respect to cognitive impairment.

## INTRODUCTION

Ginseng is a perennial plant belonging to the genus Panax and family Aralliaceae [1]. The word Panax means “all-healing” in Greek based on a view that ginseng is influential in healing all kinds of disease [2]. Among the different species of ginseng, the most commonly investigated species are *Panax ginseng* (frequently called as just ginseng or Korean ginseng), *P. quinquefolius* (also known as American ginseng) and *P. notoginseng* (also known as Chinese ginseng or Sanchi) [3]. Ginseng roots, stems and leaves have been used in traditional herbal medicine for over 2000 years [4]. Ginseng and its active components have exhibited a wide range of characteristics, including antioxidant, antiaging, antifatigue, adaptogenic, restorative, vasodilatory, anti-inflammatory, immunomodulatory, anticancer and antidiabetic [2, 4]. In addition, in models of neurological disorders, the active components of ginseng showed anti-depressive, antistress, antiepileptic and antipsychotic activities [5–8].

Cognitive health is a major factor in the life of the elderly and in preserving the ability to function. Vital elements of cognitive health include mental abilities and acquired skills, along with the aptitude to apply them so as to participate in a specific activity [9]. Age-related decline of cognitive functions is a leading challenge in mental health research. Because no curative treatment for dementia currently exists, an alternative would be to find way to attenuate cognitive impairment in older people, which, in turn, could delay the onset of dementia [10]. Moreover, in many neurological disorders, the cognitive functions of patient might be affected. For example, Alzheimer’s disease (AD) is the most common cause of dementia and is characterized by memory loss and cognitive impairment [11, 12]. Cognitive functions can be impaired in other neurological disorders such as Parkinson’s disease, multiple sclerosis and stroke [13–15].

Shi-Zhen Li recorded anxiolytic, antidepressant and cognition-enhancing effects of ginseng in the most comprehensive pre-modern herbal text (Ben Cao Gang Mu), which was compiled during the Ming Dynasty in China [15]. Recent research shown that ginseng and its active components possible have effects against neurodegenerative diseases and stroke [16–19]. Additionally, in a mechanistic study using rat model [20], saponins from *Panax japonicus* (also known as Japanese ginseng) attenuated age-related neuroinflammation by regulating signaling pathways of mitogen-activated protein kinase (MAPK) and nuclear factor kappa-light-chain-enhancer of activated B cells (NF-κB). The compound Schisandra-Ginseng-Notoginseng-Lycium produced learning and memory enhancement in scopolamine-induced learning and memory loss in mice [21]. Moreover, the recently discovered protopanaxadiol derivative 1-(3,4-dimethoxyphenethyl)-3-(3-dehydroxyl-20(*S*)-protopanaxadiol-3b-yl)-urea showed pharmacological activity in an AD model. This compound improved the daily activities and decreased cognitive deficits in APP/PS1 transgenic mice. It also reduced amyloid beta (Aβ) production mainly by inhibiting the protein kinase R (PKR)-like endoplasmic reticulum kinase (PERK) and eukaryotic initiation factor 2 (eIF2) signal-mediated beta-site APP-cleaving enzyme 1 translation. The protopanaxadiol derivative also stimulated Aβ clearance by promoting autophagy as a phosphatidylinositol-3 kinase (PI3K) inhibitor via PI3K/protein kinase B (Akt)/ mammalian target of rapamycin (mTOR) signaling pathway, while exhibiting a neuroprotective effect involving attenuation of endoplasmic reticulum (ER) stress [22]. In our recent article, we show that the active constituents of ginseng produce their effects in neurodegenerative diseases by modulating various ion channels and molecular signaling pathways [23]. Considering the importance of and recent evidence about ginseng, herein, we discuss the potential effects of active ginseng components on cognitive impairment and their fundamental chemistry and pharmacokinetics. In addition, we discuss prospects for their delivery and their use in clinical studies of cognitive impairment.

## BASIC CHEMISTRY AND CLASSIFICATION OF ACTIVE GINSENG COMPONENTS

Ginseng root contains several constituents, including triterpene saponins, polysaccharides, peptidoglycans, nitrogen-containing compounds, fatty acids, carbohydrates, phenolic compounds, and essential oil-containing polyacetylenes and sesquiterpenes [24].

The major active components in ginseng are ginsenosides, which are an arrangement of triterpene glycosides (saponins). Ginseng also contains gintonin, a nonsaponin compound [25]. Aamong the 150 ginsenosides isolated from ginseng, 40 are present in *P. ginseng* [26]. Ginsenosides contain a four-ring steroid backbone structure [27, 28], and are classified into three groups according to their aglycone structures, namely, oleanane, protopanaxadiol (PPD), and protopanaxatriol (PPT). Ginsenoside Ro is the only member of the oleanane group and contains the oleanolic acid aglycone [29]. Two major groups of ginsenosides are PPD, which includes Rb1, Rb2, Rc, Rd, Rg3, Rh2, and Rh3 and PPT, which includes Re, Rf, Rg1, Rg2, and Rh1 [30].

**Figure 1:**
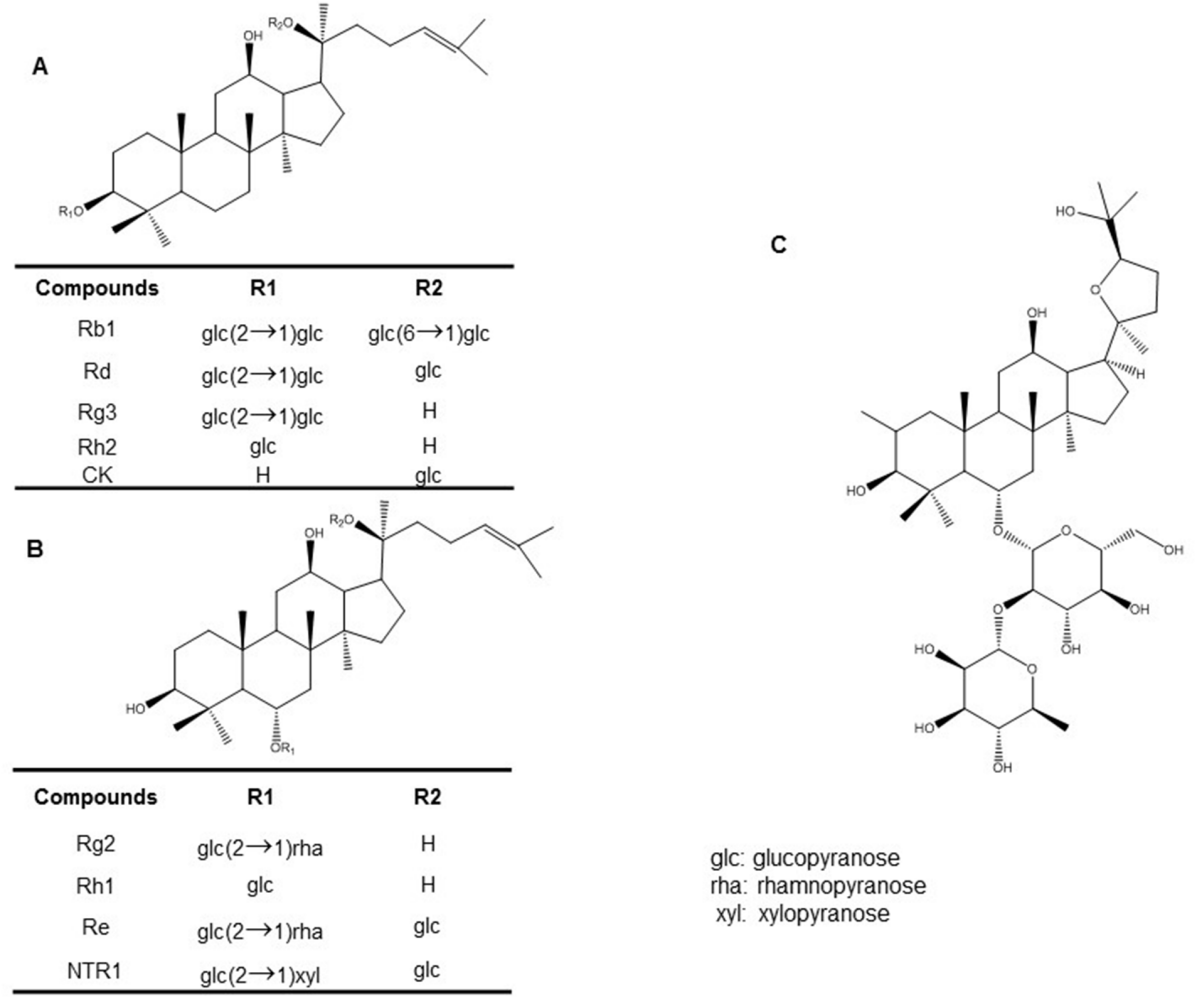
Chemical structures of various active ginseng components: **(A)** protopanaxadiol type ginsenosides **(B)** protopanaxatriol-type ginsenosides **(C)** pseudoginsenoside-F11.

Both PPD and PPT of ginsenosides exist in glycosylated compounds that contain a non-sugar component with one to four glycoside molecules [29]. Wild ginseng comprises more than 80% glycosylated major ginsenosides, including Rb1, Rb2, Rc, Rd, Rg1 and Re [31]. Furthermore, pseudoginsenoside-F11 (PF11), an ocotillol-type ginsenoside (saponin), is found in *P. quinquefolius* [32, 33]. PF11 is also known as a novel partial peroxisome proliferator-activated receptor γ (PPARγ) agonist, which could be developed into a new PPARγ-targeted therapeutic drug against type 2 diabetes [33]. Notoginsenoside R1 (NTR1), a novel phytoestrogen isolated from *P. notoginseng*, belongs to the PPT group and a major component in *P. notoginseng* [34–37]. Compound K [20-O-β-D-glucopyranosyl-20(S)-protopanaxadiol] first isolated from soil bacteria and is a YSB-6-mediated hydrolysate of a mixture of Rb1, Rb2, and Rc [38]. It is a metabolic product of Rb1 and Rb2 metabolized by intestinal bacteria [38, 39] through specific metabolic pathways [40]. The chemical structures of various active compounds of ginseng that have been studied with respect to cognitive impairment are shown in Figure 1.

## PHARMACOKINETICS OF ACTIVE GINSENG COMPONENTS

The pharmacokinetics of different active ginseng compounds has been studied in both animals and humans. After oral administration, the level of test compounds are high in the plasma sample, but the rate of absorption of ginsenosides is low [41]. Ginsenosides are not easily absorbed because of their hydrophilicity [42]. The energy-dependent absorption profile of ginsenosides in the mucosa of intestine [43–45], and the availability of both intact ginsenosides and their metabolites from the intestines are very low [39, 46, 47]. Gintonin can be absorbed into the intestine via transcellular and paracellular diffusion and the active transport process [48]. In general, the time for ginsenosides to reach maximum concentration (Tmax) in plasma is less than 2 hr, signifying that saponins are quickly absorbed and readily distributed in tissues [49, 50]. According to quantitative and statistical analyses, the plasma level of ginsenosides indicates that PPD ginsenosides have a higher concentration and longer half-life than do PPT ginsenosides [51]. After oral administration of Rb1 (only compound K) was found in plasma and urine [52]. After dosing at different time intervals, ginsenosides enter into the brain rapidly, but the concentrations rapidly decline with time. The ginsenosides with higher concentrations in the brain are Rg1, Re, Rb1 and Rc that was shown by a pharmacokinetic study [51]. Rg1 and Re are better distributed in the brain and are considered the main components that directly affect central nervous system (CNS) neurons. Conversely, they are in the circulation a long time, PPD ginsenosides may protect the brain mostly via a peripheral effect. The disposition in the liver and bile tissue confirms that cleared ginsenosides are cleared from circulation [53–55].

After the biotransformation of ginsenosides, microbiota in the gut forms deglycosylated products [55]. The absorbable metabolite compound K forms after biotransformation by microorganisms or specific enzymes. Several studies indicated that intestinal bacteria isolated from human feces or fungi derived from soil around ginseng roots as well as some food-derived microorganisms hydrolyze ginsenosides to produce compound K [56, 57]. The deglycosylated products are better able to permeate and be absorbed than ginsenosides [58]. However, the extensive of the deglycosylated products through active transport shorten the biological half-life, resulting in low systemic exposure [55]. In addition, studies that used co-administration with adrenalin [59] or lipid-based formulations [59, 60] and of the suppression of pglycoprotein efflux system [43] confirmed the increase in oral bioavailability of ginsenosides. Ginsenoside was metabolized by the hepatic cytochrome P450 3A4 (CYP3A4), which is catalyzed the metabolism by oxygenation of the hepatic disposition of ginsenosides [43]. In addition, attachment of additional sugar moieties to the PPD ginsenosides Ra3, Rb1, Rc and Rd blocks their access to biliary transporters and reduces biliary excretion [55], and furthermore, the active transport carried out by ginsenosides and their deglycosylated products for excretion by the biliary system [55]. Time curves of ginsenosides exhibited distinct multiple peaks after oral administration that demonstrating the involvement of enterohepatic recirculation [53]. Approximately, 0.2–1.2% of ginsenosides were excreted in human urine [61], with the elimination half-lives (*T*_*1/2*_) of Rg1, Re and Rb2 between 0.8 and 7.4 h in rabbits [62]. In humans, the T1/2 of the tested ginsenosides was generally less than 24 h [63, 64].

## PHARMACOLOGICAL POTENTIAL OF ACTIVE GINSENG CONSTITUENTS IN MODEL STUDIES OF COGNITIVE IMPAIRMENT

### Ginsenoside Rb1

**Figure 2:**
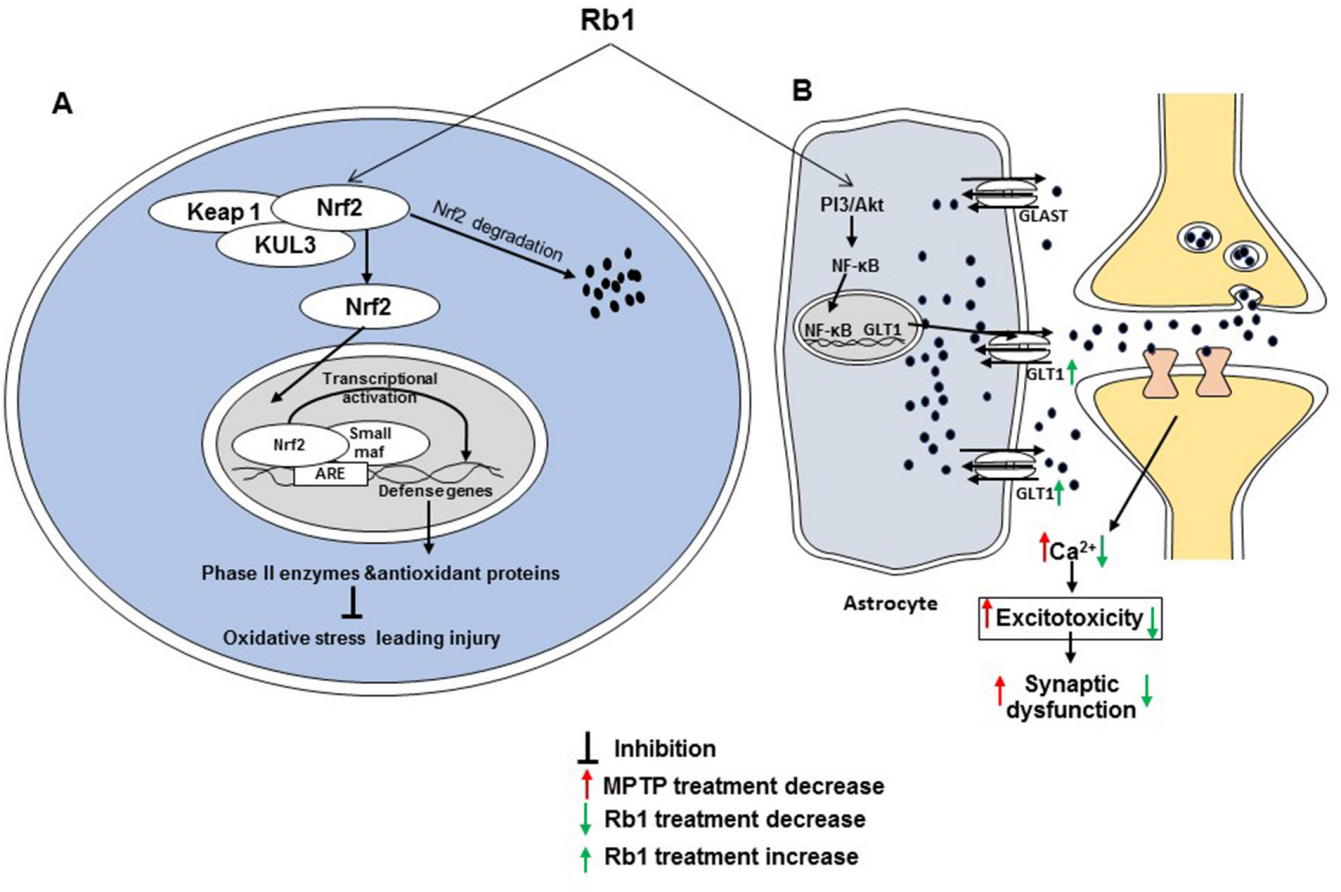
Two major activities of Rb1 associated with cognitive impairment **(A)** Rb1 prevents oxidative stress leading to damage by activating the Nrf2 pathway. **(B)** Rb1 decreases excitotoxicity-mediated synaptic dysfunction by upregulating GLT 1 via the PI3K/Akt and NF-κB pathway.

In recent studies, Rb1 produced cognitive enhancing activity in both cisplatin- and isoflurane and surgery-induced-cognitive impairment rodent models [65, 66]. In the cisplatin-induced impairment model, administration of Rb1 (2 mg/kg for 5 consecutive weeks) effectively ameliorated cisplatin-induced memory impairment in behavioral studies. Rb1 also attenuated cisplatin-induced neuronal loss in the CA1, CA3, and dentate gyrus of the hippocampus, rescued the cholinergic neuron function, and inhibited the oxidative stress and neuroinflammation in the brains of cisplatin-induced rats [65]. In the isoflurane and surgery-induced cognitive impairment model, Rb1 [60 mg/kg, intraperitoneal (i.p.) from 7 days before surgery] attenuated cognitive impairment model and synaptic dysfunction. It also mitigated the elevated levels of isoflurane and surgery-induced reactive oxygen species (ROS), tumor necrosis factor-α (TNF-α) and interleukin (IL)-6 in the hippocampus of mice [66]. In subarachnoid hemorrhage-induced brain injury in rats, Rb1 (20 mg/kg) reduced brain edema, enhanced neurobehavioral function, and blocked vasculature thickening and spasms [67]. In another recent study, Rb1 treatment ameliorated alteration of leptin-pJAK2-pSTAT3 signaling and leptin-induced brain derived neurotrophic factor (BDNF) expression in the prefrontal cortex of obese mice and improved hyperleptinemia. It also promoted the effect of leptin on neurite branching and elongation and synaptogenesis in prefrontal cortical neurons [68]. Increasing cell survival in the hippocampus may be one way in which Rb1 helps spatial learning and memory, as described in a molecular study [69]. In a comparative study between Rg1 and Rb1, the latter facilitated learning and memory in a scopolamine-induced mice model. It increased acetylcholine (ACh) levels in the hippocampus and prevented the decrease in the level of scopolamine-induced 5-hydroxytryptamine (5-HT). However, Rb1 has less of an effects than Rg1 [70]. In another comparative study and a specific study on Rb1, Rb1 attenuated *tert*-butylhydroperoxide toxicity in the neural progenitor cells, where the nuclear factor (erythroid-derived 2)-like 2 (Nrf2)/heme oxygenase-1 pathway was found to be crucial in the intracellular defense against oxidative stress [71, 72]. In addition, a recent study demonstrated the neuroprotective activity of Rb1 in 1-methyl-4-phenyl-1,2,3,6-tetrahydropyridine (MPTP)-induced Parkinson’s disease model. Rb1 treatment ameliorated MPTP-induced motor impairment, protected dopaminergic neuron death, and suppressed α-synuclein expression and astrogliosis in mouse. It increased glutamate transporter expression through nuclear translocation of NF-κB, regulated glutamate receptor expression, and promoted synaptic protein expression [73]. Two major pharmacological effects of ginsenoside Rb1 on cognitive impairment are shown in Figure 2.

### Ginsenoside Rd

Ginsenosides Rd has the potential for treating cognitive impairment in several model studies. In an amyloid β-protein precursor (APP) transgenic (Tg) mice model, Rd improved learning and memory probably via inhibiting the transcription activity of NF-κB. Suppression of the transcription activity of the NF-κB pathway might have led to the reduction of proinflammatory cytokines, and the generation of protective factors eventually increased [74]. In addition, Rd enhanced the learning and memory functions of ovariectomized rats by activating estrogen-like activity through the estrogen receptor. In HT22 cells, Rd upregulated an increase in the level of secreted amyloid precursor protein-α (sAPPα) to 10 μM, which can be inhibited by inhibitors MAPK and PI3K pathways. Estrogen receptor inhibitors prevent Rd triggered release of sAPPα and activation of MAPK and PI3K pathways. However, Rd increases expression of α-secretase and sAPPα, decreases expression of the β-secretase and Aβ, and promotes phosphorylation of estrogen receptor alpha at the Ser118 residue [75]. In a trimethyltin (TMT)-induced neurotoxicity model, pretreatment with 20 μg/ml Rd for 24 h reversed the toxic action. Notably, Rd attenuated the tremor seizures and cognitive decline in behavioral tests and significantly reduced neuronal loss (*P*=0.018) and active astroglials (*P*=0.003) compared with the negative control group. The mechanism involved in prevention of TMT-induced cell apoptosis by Rd treatment is the regulation of B-cell lymphoma 2 (Bcl-2), Bcl-2-like protein 4 (Bax) and caspase-3 [76]. Moreover, Rd may promote neurite outgrowth in PC12 cells by upregulating growth-associated protein-43 expression through both extracellular signal-regulated kinase (ERK)- and adhesion related kinase-dependent signaling pathways [77].

### Ginsenoside Re

Different studies have postulated about the ginsenoside Re activity in cognitive impairment models. Re (2 μg/ml) produced protective activity via the phospho-p38, iNOS, and COX-2 signaling pathways in lipopolysaccharide (LPS)-induced BV-2 microglial cells [78]. It improves the learning and memory in rats, via a mechanism that may correlate with its enhancing basic synaptic transmission and increasing the magnitude of the long-term potentiation (LTP) of the dentate gyrus in a rat model [79]. In addition, ginsenoside Re (10, 20, or 50 mg/kg i.p.) administered during the repeated immobilization stress period significantly inhibited the stress-induced impairment in behavioral tests. It also significantly inhibited the increase tyrosine hydroxylase (TH) expression in the locus coeruleus and the decrease BDNF mRNA expression in the hippocampus [80]. Moreover, Re shows an effect on cognitive function, oxidative stress, and inflammation in streptozotocin-induced diabetic rats. Treatments with Re (40 mg/kg) for 8weeks remarkably attenuated diabetes-associated cognitive decline, which confirmed the involvement of oxidative stress and inflammation in the development of cognitive impairment caused by diabetes [81]. In addition, Re ameliorated high-fat-diet-induced insulin resistance in C57BL/6 mice. It improved cognitive dysfunction in diabetic mice, as shown by behavioral studies and regulated ACh, acetylcholinesterase (AChE), malondialdehyde, superoxide dismutase (SOD), and oxidized glutathione (GSH)/total GSH by regulating the c-Jun N-terminal protein kinase (JNK) pathway. Therefore, Re could be used to improve high-fat diet-induced insulin resistance by ameliorating hyperglycemia via protecting the cholinergic and antioxidant systems in the mouse brain [82]. In the myocardial ischemia-reperfusion injury rat model, Re has a protective effect, the mechanism of which may be related to the alleviation of damage caused by oxygen free radicals [83]. In a pharmacokinetics study, Re dose-dependently increased the extracellular levels of dopamine and ACh in the hippocampus and medial prefrontal cortex, although it had a greater effect in the hippocampus [84]. In addition to the aforementioned models, Re also protects against phencyclidine (PCP)-induced behavioral changes and mitochondrial dysfunction. Treatment with Re significantly attenuates PCP-induced neurotoxic changes, and sociability deficits and recognition memory impairment. Modulation of the interaction between glutathione peroxidase (GPx) activity and PHOX (p47phox) is necessary for Re to exhibit its neuroprotective potential against PCP insult [85].

### Ginsenoside Rg1

Various molecular studies have researched the pharmacological potential of ginsenoside Rg1 in cognitive impairment models. Rg1 treatment (30 mg/kg i.p. for 10 days) improved chronic morphine-induced spatial learning capacity and restored the morphine-inhibited LTP. These effects were depended on N-methyl-d-aspartic acid (NMDA) receptor [86]. In a scopolamine-induced memory and learning impairment study, Rg1 (6 and 12 mg/kg i.p. for 7 days) improved ACh levels and inhibited AChE activity in the hippocampus and prevented the decrease of scopolamine-induced *5*-*HT* [70]. In a D-galactose-induced impairment rat model, Rg1 treatment (20 mg/kg i.p. for 28 days) displayed activity against memory impairment. It possibly attenuated all the D-galactose-induced changes in the hippocampus, such as cognitive capacity, senescence-related markers, and hippocampal neurogenesis, compared to those that occurred in the untreated rats. Further investigation showed that Rg1 protects neural stem cells/neural progenitor cells (NSCs/NPCs) as indicated by the elevated SOX-2 expression level, and reduces astrocyte activation, as indicated by the reduced astrocyte elevated gene-1 expression level. It also increases hippocampal cell proliferation and enhances the activity of the antioxidant enzymes GPx and SOD. Rg3-treatment decreased proinflammatory cytokines such as IL-1β, IL-6, and TNF-α, but, increased the telomere lengths and telomerase activity and downregulated the mRNA expression of cellular senescence-related genes p53, p21Cip1/Waf1, and p19Arf in the hippocampus of aged rats [87]. A mechanistic study showed that long-term administration of Rg1 supplement improved the performance of aged mice in a behavior test and significantly upregulated the expression of hippocampal synaptic plasticity-associated proteins such as synaptophysin, NMDA receptor subunit 1 (GluN1), postsynaptic density-95, and calcium/calmodulin-dependent protein kinase II alpha by promoting the activation of mTOR pathway [88]. In a chronic restraint stress (CRS) rat model, Rg1 ameliorated cognitive deficits, especially the loss of adaptation capacity. CRS decreases the levels of BDNF, tropomyosin receptor kinase B (TrkB), and ERK phosphorylation in the prefrontal cortex of CRS rats. However, these changes were effectively reversed by Rg1 (5 and 10 mg/kg i.p.) [89]. Rg1 also showed remarkable effects in isoflurane-, LPS- and dexamethasone-induced cognitive impairments in rodent models. Treatment with Rg1 (20 mg/kg for 7 days) significantly improved cognitive function and exhibited antioxidant and anti-inflammatory effects, demonstrating the neuroprotective effects of ginsenoside Rg1 against the effect of isoflurane anesthesia in the rats. In addition, it significantly reduced caspase-3 activity, upregulated the expression of PI3K/protein kinase B/glycogen synthase-3β, and downregulated the mRNA expression levels of p21WAF1/CIP1 and p53 in aged rats exposed to isoflurane anesthesia [90]. It has a protective effect against LPS-induced cognitive deficit and its prevention of LPS-induced changes in the cholinergic system is crucial to this ameliorating effect [91]. In a dexamethasone-induced cognitive impairment mice model, Rg1 (2 and 4 mg/kg) treatment increased spontaneous motor activity and exploratory behavior in an open field test and increased the number of entries into the new object zone in a novel object recognition test. Moreover, Rg1 (2 and 4 mg/kg) treatment significantly alleviated neuronal degeneration and increased microtubule-associated protein 2 expression in the frontal cortex and hippocampus. Inhibition of NACHT, LRR and PYD domains-containing protein (NLRP)1 inflammasomes was also involved in the mechanism underlying the effect of Rg1 on dexamethasone-induced neuronal injury [92]. Rg1 improved the postoperative survival rate and protected against sepsis-associated learning and memory impairment in a sepsis-associated encephalopathy model. It attenuated histopathologic changes in the brain, suppressed Iba1 activation, decreased the expression of inflammatory cytokines, including TNF-α, IL-1β, and IL-6, and reduced neuronal apoptosis (cleaved caspase 3 activation) in the hippocampus. Study of the mechanism of these effects by Rg1 showed that it suppressed the expressions of light chain 3eII and p62 in the hippocampus [93]. Rg1 (10 mg/kg for 30 days) enhanced long-term memory in middle-aged mice. Consistent with the improvement in memory, ginsenoside Rg1 administration facilitated weak theta-burst stimulation-induced LTP in acute hippocampal slices from middle-aged mice and increased the dendritic apical spine numbers and area in the CA1 region. It is also upregulated the expression of hippocampal phospho-Akt, BDNF, proBDNF and glutamate receptor 1 (GluA1). Interestingly, the phosphatase and tensin homolog deleted from the chromosome 10 inhibitor bpV mimicked the effects of ginsenoside Rg1, including increasing p-Akt expression, and promoting hippocampal basal synaptic transmission, LTP, and memory [94]. Rg1 (10 mg/kg) suppressed the spontaneous neuronal activity of approximately 50% of the recorded-pyramidal cells in the medial prefrontal cortex. In addition, Rg1 attenuated LTP in the hippocampal–medial prefrontal cortical pathway in a rat model [95]. In addition to its use in various toxic agents-induced impairment models, Rg1 produced protective and cognitive-enhancing activities in a transgenic mouse model of AD, which was constructed via the overexpression of APP and presenilin 1. Mice were treated with 0.1-10 mg/kg of Rg1 via intraperitoneal injection. Rg1 treatment (10 mg/kg for 30 days) improved memory. As demonstrated by biochemical experiments, Rg1 reduced the accumulation of Aβ_1-42_ and phosphorylated (p)-Tau in the AD model. BDNF and p-TrkB synaptic plasticity-associated proteins were upregulated following administration of Rg1. Correspondingly, LTP was restored following administration of Rg1 in the AD mouse model [96]. In a molecular study using 3xTg-AD mice, Rg1 improved memory and ameliorated depression-like behaviors. Proteomic analysis revealed that downregulation of complexin-2, synapsin-2, and synaptosomal-associated protein 25 in the hippocampus of 3xTg-AD mice was significantly greater than that in compared with the WT mice, and Rg1 treatment modulated the expression of complexin-2 and synaptosomal-associated protein 25 in the hippocampus of 3xTg-AD mice [97]. The potential therapeutic activities of ginsenoside Rg1 in various models of cognitive impairment are presented in Table 1.

**Table 1:** Activity of ginsenoside Rg1 in different models of cognitive deficits

Model	Dose	Key effects	References
Morphine-induced memory impairment in rats	30 mg/kg i.p. for 10 days	Improves spatial learning capacity and restores LTP, and effect was NMDA receptor dependent	[86]
Scopolamine-induced memory deficits in mice	6 and 12 mg/kg, i.p. for 7 days	Improves ACh levels and inhibits AChE activity in the hippocampus; prevents the decrease of scopolamine-induced 5-HT	[70]
D-Galactose-induced memory impairment in rats	20 mg/kg i.p., for 28 days	Protects NSCs/NPCs by elevating SOX-2 expression level; reduces astrocyte activation indicated by decreasing Aeg-1 expression level; increases the hippocampal cell proliferation and enhances the activity of the antioxidant enzymes GPx and SOD; decreases the pro-inflammatory cytokines but increases telomere lengths and telomerase activity; down-regulates mRNA expression of cellular senescence related genes p53, p21Cip1/Waf1 and p19Arf in hippocampus of aged rats	[87]
Age-Related Cognitive Decline in C57BL/6J mice	6 mg/kg p.o. every third day	Upregulates expression of hippocampal synaptic plasticity-associated proteins such as synaptophysin, N-methyl-D-aspartate receptor subunit 1, postsynaptic density-95, and calcium/calmodulin-dependent protein kinase II alpha, through promoting the activation of mTOR pathway	[88]
Chronic restraint stress in rat	5 and 10 mg/kg i.p. for 24 days	Ameliorates decrease in levels of BDNF, TrkB and ERK phosphorylation in prefrontal cortex	[89]
Isoflurane-induced memory impairment in rat	20 mg/k, i.p. for 7 days	Reduces caspase-3 activity; upregulates the expression of PI3K/Akt/GSK-3β; downregulates mRNA expression levels of p21WAF1/CIP1 and p53	[90]
LPS-induced cognitive deficit in rat	200 mg/kg for 30 days	Prevents decrease in ACh levels and increase of AChE activity; revert decrease of α7 nAChR protein expression in prefrontal cortex and hippocampus	[91]
Dexamethasone-induced memory impairment in mice	2 and 4 mg/kg p.o. for 28 days	Increases expression of glucocorticosteroid receptor and decreases expression of NLRP1, ASC, caspase-1, caspase-5, IL-1β and IL-18 in hippocampus	[92]
Sepsis-associated encephalopathy in mice	40 and 200 mg/kg 10 ml/kg i.p.1 h before operation	Attenuates brain histopathologic changes, suppresses Iba1 activation; decrease expression of inflammatory cytokines, including TNF-α, IL-1β, and IL-6; reduces neuronal apoptosis (cleaved caspase 3 activation) in hippocampus; suppresses the expressions of light chain 3-II and p62 in hippocampus	[93]
Theta-burst stimulation-induced LTP in mice	0.1, 1 or 10 mg/kg i.p. once a day for 30 consecutive days	Facilitates hippocampal basal synaptic transmission and LTP; up-regulates hippocampal BDNF and p-Akt expression; increases hippocampal dendritic spines	[94]
LTP in rat	1, 3, or 10 mg/kg systemic administration	Impairs LTP in HP–mPFC pathway, perhaps by suppressing the firing of a subset of mPFC neurons	[95]
Overexpression of APP and PS1 in mice	0.1-10 mg/kg i.p. for 30 days	Repairs hippocampal LTP and memory, likely through facilitating the clearance of AD-associated proteins and activation of BDNF-TrkB pathway	[96].
3xTg-AD mice	20 mg/kg i.p. for 6 weeks	Improves behavioral deficits in AD via modulating expression of proteins (i.e., CPLX2, SYN2, and SNP25).	[97]

### Ginsenosides Rg2 and Rg3

In a vascular dementia (VM) rat model, ginsenoside Rg2 protected against memory impairment via an anti-apoptotic mechanism. VM decreased expression of Bcl-2 and the heat shock protein 70, while increasing Bax and P53. After ginsenoside Rg2 (2.5, 5 and 10 mg/kg) treatment, the expression of Bcl-2 and heat shock protein 70 increased and Bax and P53 decreased compared with the expression levels in the VM-only group [98].

Ginsenoside Rg3 had effects in both scopolamine- and LPS-induced memory impairment in models [99–101] In the scopolamine-induced memory impairment mouse model, Rg3 [50 and 100 mg/kg, per oral (p.o.)] suppressed the scopolamine-mediated increase in AChE activity and stimulation of the NF-κB pathway (i.e., phosphorylation of p65) in the hippocampus [100]. In addition, administration of Rg3 (10, 20, and 50 mg/kg, i.p.) for 21 consecutive days markedly reduced the LPS-induced learning and memory impairment in rat behavioral studies. It significantly decreased the expression of proinflammatory mediators such as TNF-α, IL-1β, and COX-2 in the hippocampus [99]. In a recent study, Rg3 prevented H_2_O_2_-induced astrocytic senescence and ameliorated the paracrine effects of senescence on glioblastoma. Rg3 (5 μg/ml) effectively prevented the astrocytic senescence induced by H2O2 exposure. Rg3 treatment at 10 μg/ml effectively suppressed the expressions of IL-6 and IL-8, which is associated with regulation NF-κB and p38MAPK activation. In addition, after incubation with Rg3, the conditioned medium from senescent astrocytic CRT cells significantly decreased the ability to promote the proliferation of astrocytoma U373-MG, U87-MG, and U251-MG cells compared with nontreated senescent samples. Similar patterns were confirmed in chemotherapy-induced senescent glioblastoma cells [102]. Additionally, Rg3 restored proliferation and inhibited apoptosis by altering the cell cycle in NMDA-treated HT22 murine hippocampal neuronal cells. It improved the change in physiological behavior in a chronic mild stress model, as seen in forced swim, tail suspension, and sucrose preference tests. The effects were facilitated by the phosphorylation of cAMP response element-binding protein (CREB) and BDNF signaling [103]. The activity of biodegradable poly(lactic-co-glycolic acid)-ginsenoside Rg3 nanoformulation in an AD model was described in a recent article [104]. This nanoformulation reduced Aβ plaques and downregulated β-amyloid A4 precursor protein (AβPP-A4) gene expression levels and produces neuroprotection. The neuroprotective activity of this novel formulation results from the prevention of oxidative stress and mitochondrial dysfunction.

### Ginsenosides Rh1, Rh2 and Rh3

In a scopolamine-induced cognitive impairment rodent model, ginsenoside Rh1 and Rh3 have a negative effect against impairment. In rat behavioral studies, Rh1 (5 and 10 mg/kg) and Rh3 (10 mg/kg) ameliorated scopolamine-induced memory impairment [105, 106]. Rh1 increased hippocampal excitability in the dentate gyrus [105]. It significantly enhanced cell survival in the dentate gyrus of mice and upregulated BDNF expression [107]. In a recent study, Rh1 had a beneficial effect against sleep deprivation-induced cognitive impairment in mice. In addition, the ability Rh1 (20 μM/kg and 40 μM/kg for 23 days) reduce oxidative stress in the cortex and hippocampus that might be involved in the mechanism of action [108]. Moreover, Rh2 (10 mg/ml for 3 weeks) significantly improved spatial learning and memory and promoted cell survival and genesis [109]. Like Rh1 and Rh2, ginsenoside Rh3 has a protective effect against cognitive impairment and also inhibits AChE activity in a dose-dependent manner. Besides, Rh3 also reversed hippocampal BDNF expression and reduced the phosphorylation of CREB in scopolamine-induced cognitive impairment mice [106].

### Pseudoginsenoside-F11

PF11 produces cognitive-enhancing activities in both scopolamine- and morphine-induced cognitive impairment rodent models. PF11 (2 and 4 mg/kg) antagonized scopolamine-induced memory dysfunction in both mice and rats. In addition, in the morphine-induced cognitive impairment mice model, PF11 (4 and 8 mg/kg) improved the behavioral responses of mice in the Morris water maze test and antagonized the development of analgesia tolerance to morphine in the tail pinch test [110, 111]. A recent mechanistic study showed that PF11 (8mg/kg) inhibited METH-induced hyperlocomotion, preference, and an increase in accumbal extracellular dopamine by regulating GABAergic neurons and μ-opioid receptors [112]. Moreover, PF11 produced cognitive enhancing effects in both Aβ_1-42_-induced and Tg-APPswe/PS1dE9 (APP/PS1) mouse AD models and in 6-hydroxydopamine (6-OHDA)-induced Parkinson’s disease model [113, 114]. In AD mouse models, PF11 treatment at doses of 1.6 and 8 mg/kg for 15 days significantly mitigated Aβ_1-42_-induced learning and memory impairment and PF11 treatment at a dose of 8 mg/kg for 4weeks 8 mg/kg dose for 4 weeks improved memory in APP/PS1 mice in behavioral studies. In the APP/PS1 mice, PF11 significantly inhibited the expressions of APP and Aβ_1-40_ in the cortex and hippocampus, restored the activities of SOD and GPx, and decreased the production of malondialdehyde in the cortex. It also noticeably improved the histopathological changes in the cortex and hippocampus and downregulated the expressions of JNK2 p53 and cleaved caspase 3 in the hippocampus [113]. In a 6-OHDA-induced PD rat model, PF11 (3, 6, and 12 mg/kg) markedly improved the locomotion, motor balance, coordination, and apomorphine-induced rotation skills in rats. PF11 treatment increased the expression of TH in the substantia nigra and also significantly increased the content of extracellular dopamine in the striatum. Compared with the 6-OHDA-lesioned group, PF11 treatment significantly reduced in the levels of striatal extracellular hydroxyl radical (∙OH), detected as 2,3- and 2,5-dihydroxy benzoic acid (DHBA), and increased the level of striatal extracellular ascorbic acid [114]. Furthermore, PF11 had a neuroprotective effect against permanent middle cerebral artery occlusion (pMCAO) in rats. One 12 mg/kg dose of PF11 significantly decreased the infarct area, reduced brain water content, and improved neurological functions, even 4 h after the onset of pMCAO. PF11 lessened the ischemic insult-mediated loss of neurons and the activation of astrocytes and microglia and it attenuated pMCAO-induced accumulations of autophagosomes and apoptosis. In addition, PF11 had a remarkable effect in reversing the ischemic insult-induced accumulation of autophagosomes (LC3-II) and abnormal aggregation of autophagic proteins (SQSTM1 and ubiquitin). It was capable of improving lysosomal function and lysosome/autophagosome fusion following pMCAO. This change was reversed by the lysosomal inhibitor chloroquine, which, in turn reversed improvement in ischemic outcome and the antiapoptotic effect [115].

### Notoginsenoside R1

**Figure 3:**
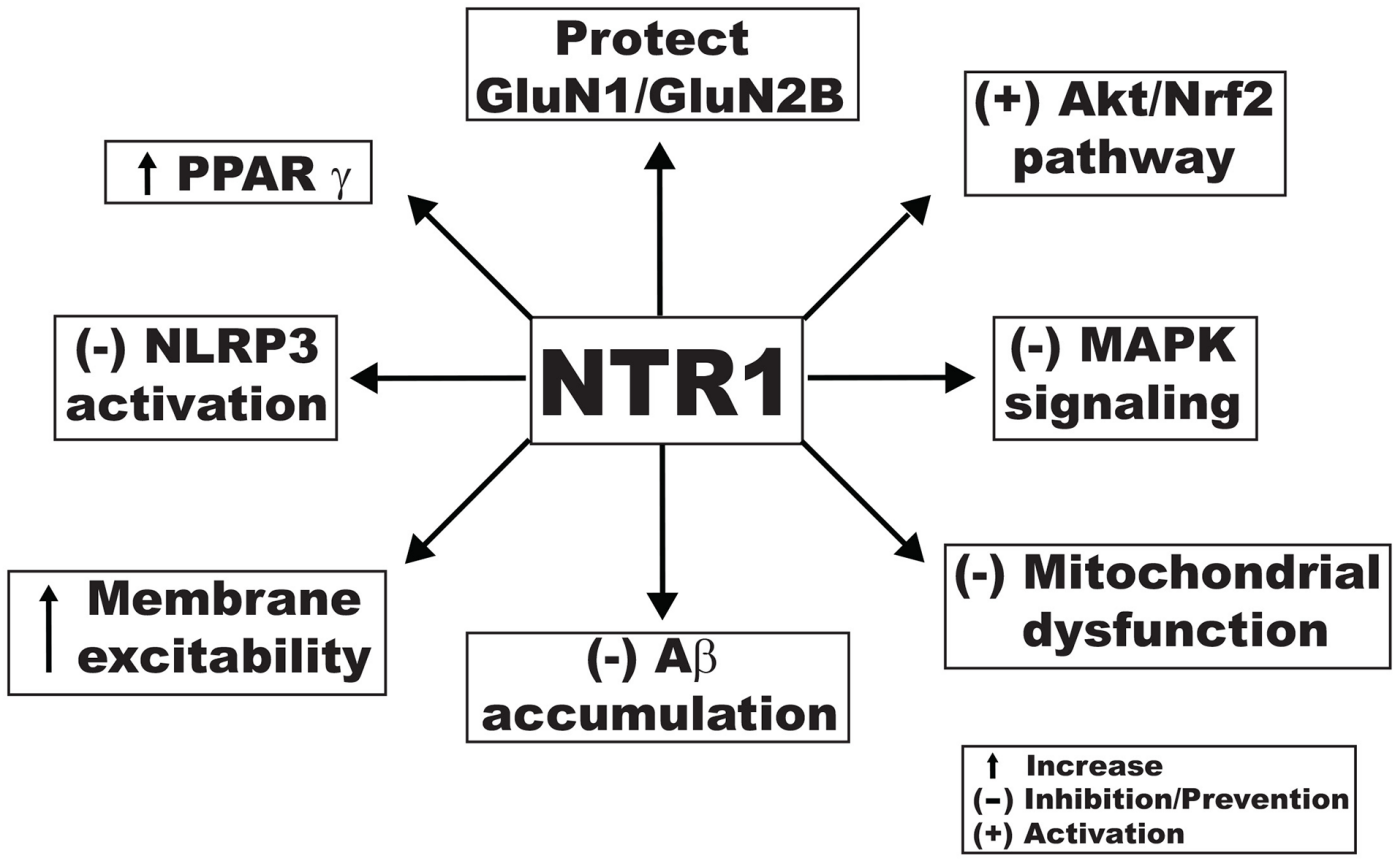
Proposed mechanistic activities of notoginsenoside R1 against cognitive impairment produced through different signaling pathways

Several molecular studies have noted the effect of notoginsenoside R1 (NTR1) on cognitive impairment. Treatment with 10 μM NTR1 protected cells expressing the NR1/NR2B subunit from the NMDA-induced cell death in primary cultured mouse cortical neurons [116]. In an Aβ-induced AD model, 10 μM dose significantly counteracted the effects of Aβ by increasing cell viability, reducing oxidative damage (including apoptosis), restoring mitochondrial membrane potential, and suppressing stress-activated MAPK signaling pathways [117]. In another study, NTR1 increased the membrane excitability of CA1 pyramidal neurons in hippocampal slices by lowering the spike threshold possibly via a mechanism that involves the inhibition of voltage-gated K^+^ currents. NTR1 also reversed Aβ_1-42_-induced impairments in LTP. Reduction of spontaneous firing activity with 10 nM tetrodotoxin abolished the protective effect of NTR1 against Aβ-induced LTP impairment. Finally, oral administration of NTR1 improved the learning performance of APP/PS1 mice in the AD model [118]. Oral administration of 5 and 25 mg/kg NTR1 significantly ameliorated cognitive function and increased the expression of choline acetyl transferase, as compared to vehicle-treated mice. Furthermore, NTR1 treatment inhibited Aβ accumulation and increased the expression of insulin degrading enzyme in both APP/PS1 mice and N2a-APP695sw cells, suggesting that NTR1 may exert its protective effects through the enhancement of the Aβ degradation. It increased the level of PPARγ and the up-regulation of insulin-degrading enzyme induced by NTR1 was inhibited by administration of GW9662 (a PPARγ antagonist), indicating that the effect of NTR1 was mediated, in part, by PPARγ [119]. In a diabetic encephalopathy model, administration of 10 and 30 mg/kg NTR1 for 10 weeks ameliorated cognitive dysfunction, depression-like behaviors, insulin resistance, hyperinsulinemia, dyslipidemia, and inflammation in db/db mice. NTR1 (20 μM) markedly decreased the oxidative stress induced by hyperglycemia in hippocampal neurons. NTR1 significantly activated the Akt/Nrf2 pathways and inhibited NLRP3 inflammasome activation in hippocampal neurons, which might be essential with respect to the neuroprotective effects of NTR1. Pretreatment with the phosphatidylinositol 3-kinase (PI3K) inhibitor LY294002 negated the neuroprotective effects of NTR1 against oxidative stress and NTR1-mediated NLRP3 inflammasome activation in high glucose-treated HT22 hippocampal neurons [120]. The proposed activities of NTR1 against cognitive impairment are presented in Figure 3.

### Gintonin

In a recent study, gintonin produced activity against cognitive impairment in a mice model. Oral administration of 50 mg/kg gintonin for 1 week significantly improved behavioral functions in the contextual fear-conditioning test. Gintonin treatment increased the expression of learning and memory-related proteins such as the phosphorylated CREB protein and BDNF and gintonin administration enhanced LTP in the hippocampus [121]. In another study, gintonin played a role in sAβPPα release, Aβ formation, Swedish-AβPP transfection-mediated neurotoxicity in SH-SY5Y neuroblastoma cells, and Aβ-induced neuropathy in mice. It mediated the promotion of non-amyloidogenic processing to stimulate sAβPPα release to restore brain function in mice with AD [122]. Another report described the effect of gintonin on an Aβ-induced cognitive impairment model where by at the doses of 25 or 50 mg/ kg for 2 weeks, attenuated Aβ-induced cholinergic dysfunctions. Gintonin treatment increased ACh concentration, choline acetyltransferase activity, and immunoreactivity, but it decreased AChE activity. In addition to Aβ-induced impairment model, long-term oral administration of gintonin (25 or 50 mg/kg for 3 months) showed potential activity in a transgenic AD mouse model. It also attenuated AD-related cholinergic impairment in that model [123]. Lysophosphatidic acid (LPA) is a well-characterized and ubiquitous phospholipid molecule that produced effect by activating G protein-coupled receptors known as LPA receptors (LPARs). During the early stages of development, LPAR signaling is critically involved in the regulation of synapse formation and the morphology of cortical and hippocampal neurons. Moreover, LPARs seem to contribute to cognitive as well as emotional learning and memory in adult brains [124]. As a ligand, gintonin activates LPAR with high affinity [122, 125] and activates Ca^2+^-activated Cl channels in *Xenopus* oocytes by activating LPAR. Gintonin mediation of INMDA potentiation and LTP induction in the hippocampus via the activation of LPAR might account for ginseng-mediated improvement of memory-related brain functions [125]. Furthermore, gintonin induced [Ca^2+^]_i_ transients in cultured mouse hippocampal neural progenitor cells (NPCs). These [Ca^2+^]_i_ transients were linked to stimulation of ACh release through LPAR activation [123]. The gintonin mediated LPAR activation vastly improved both excitatory and inhibitory transmission in central synapses and resulted in synaptic enhancement and an increase in neuronal excitability in a phospholipase C-dependent manner [124]. Gintonin increased the incorporation of 5-bromo-2'-deoxyuridine (BrdU) in hippocampal NPCs in a dose- and time-dependent manner. At 0.3 μg/ml, gintonin increased the immunostaining of glial fibrillary acidic protein, NeuN, and LPA1 receptor in hippocampal NPCs. However, as an LPA1/3 receptor antagonist and Ca^2+^ chelator block, gintonin induced an increase in BrdU incorporation and immunostaining of biomarkers. Oral administration of a gintonin-enriched fraction (50 and 100 mg/kg) increased hippocampal BrdU incorporation and LPA1/3 receptor expression in adult wild-type and transgenic AD mice [126].

### Compound K

**Figure 4:**
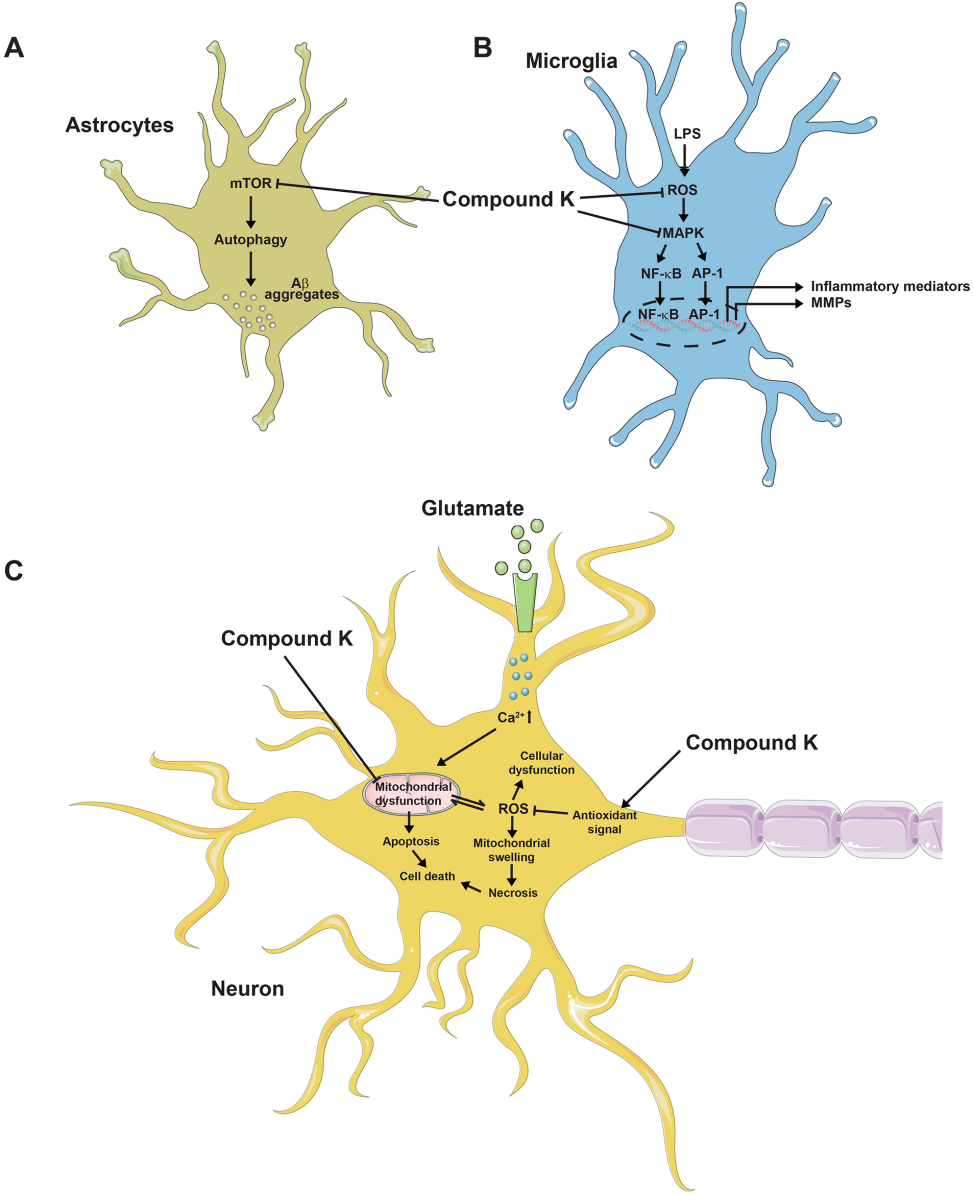
Probable activities of compound K against cognitive impairment **(A)** Compound K inhibits mTOR pathway in astrocyte, leading to autophagy-dependent Aβ clearance. **(B)** Compound K produces anti-inflammatory activity in microglia by inhibiting ROS generation and MAPK pathway. **(C)** Compound K prevents mitochondrial dysfunction and activates antioxidant signal in neurons, possibly leading to protective activity against cell death.

In the cyclophosphamide-induced cognitive impairment mice model, compound K activity acts against cognitive impairment. Cognitive deficits an adverse effect of the chemotherapeutic agent, but 4 weeks treatment with compound K (2.5, 5 and 10 mg/kg) improves memory. Moreover, compound K (10 mg/kg) ameliorates the cyclophosphamide-induced decrease of neurogenesis in the hippocampus [127]. A recent study showed that it improved memory function in cyclophosphamide-induced and glutamate-induced cytotoxicity in hippocampal HT22 cells. Compound K (8 μM) induced antioxidant enzymes in an Nrf2-mediated manner and effectively attenuated cytotoxicity and mitochondrial damage induced by glutamate in HT22 cells. However, tin protoporphyrin IX (heme oxygenase-1 inhibitor) negated the cytoprotective effect of compound K. Therefore, Nrf2-mediated induction of antioxidant enzymes might involve in neuroprotective effect of compound K. In addition, compound K (10 mg/kg) enhanced learning and memory performance in C57BL/6 mice used in a scopolamine-induced model [128]. In an LPS-induced neuroinflammation model, compound K inhibited the expression of iNOS, proinflammatory cytokines, monocyte chemotactic protein-1, matrix metalloproteinase-3, and matrix metalloproteinase-9 in both BV-2 microglial cells and primary cultured microglia. Compound K suppressed microglial activation by inhibiting the activities of ROS, MAPK, and NF-κB/activator protein-1 via the enhancement of heme oxygenase-1/antioxidant response element signaling. Based on its effect on microglia, compound K also reduces the number of Iba1-positive activated microglia and inhibits the expression of TNF-α and IL-1β in the brains with LPS-induced sepsis. Compound K also reduces the infarct volume of ischemic brain induced by pMCAO and suppresses microglial activation in the ischemic cortex [129]. In addition to its effect on microglia, compound K enhances Aβ clearance by increasing autophagy via the mTOR signaling pathway in primary astrocytes [130]. The potential activities of compound K against cognitive impairment are presented in Figure 4.

## PROSPECTS FOR DELIVERY AND CLINICAL STUDY OF GINSENG COMPONENTS FOR COGNITIVE IMPAIRMENT

Although clinical studies on ginseng have been performed, the effect of individual active ginseng components in cognitive impairment has not been studied. Of the various ginseng species, Korean and American ginsengs have been studied for their clinical efficacies. Korean ginseng increases cognitive performance in AD patients [18, 131]. One study included volunteers (*n*=58) treated with ginseng powder (4.5-g/day) for 12 weeks and untreated control group (*n*=39). The Alzheimer disease Assessment Scale cognitive subscale (ADAS-cog) and the mini-mental state examination (MMSE) score began to show improvement up to 12 weeks after ginseng treatment. The improvement in the ADAS-cog and MMSE declined to the levels of the control group after the discontinuation of ginseng treatment [131]. After confirming the cognitive benefits of *P. ginseng*’s in AD patients in a 24-week randomized open-label study, treatment continued for up to two years to further determine its long-term effect. While the subjects took 4.5 or 9.0 g *P. ginseng* per day, cognitive function was evaluated every 12 weeks using the ADAS-cog and MMSE. There was a significant improvement in both ADAS-cog and MMSE scores of the *P. ginseng*-treated groups at 24 weeks [18]. Other studies confirmed the cognitive-enhancing activity of Korean ginseng [132, 133]. In a study of healthy volunteers (*n*=15) without impairments, Korean ginseng had a cognitive-enhancing effect in the treatment group (4500 mg daily) in compared with the placebo group [132]. In another study, *P. ginseng* had a cognitive-enhancing effect in healthy volunteers (*n*=90) with mild cognitive impairments. The subjects in the ginseng-treated group showed significant improvement on the visual learning test and the visual recall test after 6 months of *P. ginseng* treatment compared with the placebo group [133]. American ginseng at 100, 200 and 400 mg also had cognitive-enhancing effects in a randomized, double-blind, placebo-controlled, crossover study of healthy young adults (*n*= 32). The working memory performance and Corsi block performance of the treated group improved. Moreover, choice reaction time accuracy and calmness significantly improved with the 100 mg treatment [134]. In another study, HT1001, a standardized proprietary *P. quinquefolius* extract containing Rb1, Rg1 and other important ginsenosides was administered (dose=200 mg) to healthy volunteers who were divided into two groups: a young adult sample (*n*=10) and a middle-aged sample (*n* =10). Study found that HT1001 might be a candidate for further clinical study on learning and memory [135].

The therapeutic potential of ginsenosides has been limited mostly by low-bioavailability that is caused by low aqueous solubility, poor bio-membrane permeability, instability in the gastrointestinal tract, and extensive metabolism in the body [136]. Several experiments using formulated ginsenoside nanoparticles have been conducted. PLGA-encapsulated ginsenoside Rg3 increased antitumor activity [137]. Bovine serum albumin (BSA)-Rh2 nanoparticle (NP) formulation has improved solubility and stability and has enhanced anticancer and anti-inflammatory effects in cell lines [138]. In addition to a nanoparticle delivery system, some of studies have focused on liposome for delivery. Compound K-loaded liposomes modified with D-α-tocopheryl polyethylene glycol 1000 succinate (TPGS) (GCKT-liposomes) have enhanced solubility and show significant antitumor activity [139]. Among the three formulations of Rh2-loaded liposomes, i.e., Rh2-loaded normal liposome, Rh2-loaded cationic liposome, and Rh2-loaded methoxy poly(ethylene glycol)-polylactide (mPEG-PLA) liposome, the latter might be promising way to enhance the therapeutic index of anticancer agents [140]. Moreover, a liposomal formulation of Rg3 might improve anticancer activity [141]. Considering the pharmacokinetic behavior, many recent studies have focused on its novel formulation and delivery. Few studies have focused on intranasal delivery of active ginseng components as a way to target the brain. Ginsenosides Rg1 and Rb1 in Nao-Qing microemulsion promoted absorption and achieved a fast effect in an acute ischemic stroke model [142]. In another study, intranasal delivery of Rb1 exerted brain-targeting effects and ameliorated ischemia/reperfusion insult immediately after MACAO. Autophagy was involved in these beneficial effects [143]. Rg1 nanoparticles were developed to overcome the blood-brain barrier (BBB)-mediated restriction of Rg1 to reach the CNS. At a high concentration, Rg1 nanoparticles can penetrate the BBB to reduce the cerebral infarction volume and promote neuronal recovery *in vivo* [144]. In addition, nanoparticle formulation of Rg3 offered neuroprotective activity in an AD model. This formulation is biodegradable and has minimal side effects giving it potential for AD therapy [104].

Setting up a clinical study of the use of different ginseng species for treating cognitive deficits is a major challenge in ginseng research. Second, conducting a clinical study on active ginseng components in a designed cognitive impairment model is recommended. Third, novel formulations of active ginseng constituents are candidates for preclinical and clinical models of cognitive impairment.

## CONCLUDING REMARKS

Ginseng has long been used as a traditional medicine in many countries because of its high therapeutic value. It displays beneficial roles against various diseases and disorders. In neurological disease model, ginseng and its active components show the potential for pharmacological activity. In the present article, we reviewed the therapeutic potential of its active constituents in cognitive impairment, along with modes of delivery and challenges concerning clinical studies. Active ginseng constituents, including ginsenosides Rb1, Rd, Re, Rg1, Rg2, Rg3, Rh1, Rh2, Rh3, PF11, and NTR1, and gintonin and compound K have shown potential activity in treating cognitive deficits. They produced effects in both cellular and animal model studies. Different animal models used for behavioral studies have been postulated to study the effects of ginseng and its active components on cognitive impairment induced by different well-known neurotoxic agents. Moreover, studies based on biochemistry, molecular biology, and histochemistry confirmed their possible therapeutic activity against cognitive impairment. In addition to animal models, several studies have focused on cellular models to prove the potential of ginseng and its active components. The studies have also shown possible pharmacological activity produced by ginseng and its components. In both animal and cellular model studies, they produced activity against oxidative stress leading to cognitive-enhancing function. Apart from oxidative stress, many active components produce anti-neuroinflammatory agents that might be associated with their activities in cognitive impairment. More importantly, our data has shown that several ginseng constituents exert their effects by modulating cholinergic, glutaminergic, and other molecular signaling pathways that are vital for cognitive activity.

Numerous recent studies have reported on the potential therapeutic efficacy of active ginseng components, but more studies utilizing novel techniques are needed. The effect of active components should be confirmed at the molecular level using a preclinical study focused on transgenic and toxin-induced wild-type animal models. In both animal and cellular model studies, knockout and knockdown animal models should be used to focus on molecular signals associated with cognition. Finally, because of the small number of studies on active ginseng components, we recommend the design and performance of experiments with ginseng constituents and their analogues in novel formulations for treating cognitive deficits at the clinical level.

## Abbreviations

5-HT: 5-hydroxytryptamine
5-HT: 5-hydroxytryptamine
6-OHDA: 6-Hydroxydopamine
ACh: Acetylcholine
AChE: Acetylcholinesterase
AD: Alzheimer’s disease
Aeg-1: Astrocyte elevated gene-1
Akt: Protein kinase B
APP: Amyloid β-protein precursor
ASC: Antibody secreting cells
Aβ: Amyloid beta
Bax: Bcl-2-like protein 4
Bcl-2: B-cell lymphoma 2
BDNF: Brain-derived neurotrophic factor
BrdU: 5-Bromo-2'-deoxyuridine
CPLX2: Complexin-2
CREB: cAMP response element-binding protein
CRS: Chronic restraint stress
ERK: Extracellular signal-regulated kinase
GPx: Glutathione peroxidase
GSH: Glutathione
GSK-3β: Glycogen synthase kinase 3
HP–mPFC: hippocampal–medial prefrontal cortex
i.p.: Intraperitoneal
Iba1: Ionized calcium-binding adapter molecule 1
IL: Interleukin
JNK: c-Jun N-terminal protein kinase
LPA: Lysophosphatidic acid
LPAR: LPA receptor
LPS: Lipopolysaccharide
LTP: Long-term potentiation
MAPK: Mitogen-activated protein kinase
mTOR: Mammalian target of rapamycin
NF-κB: Nuclear factor kappa-light-chain-enhancer of activated B cells
NLRP: NACHT, LRR and PYD domains-containing protein
NMDA: *N*-methyl-D-aspartic acid
Nrf2: Nuclear factor (erythroid-derived 2)-like 2
NSCs/NPCs: Neural stem cells/neural progenitor cells
NTR1: Notoginsenoside R1
p-Akt: Phospho-Akt
p.o.: Per oral
PF11: Pseudoginsenoside-F11
PI3K: Phosphatidylinositol-3 kinase
PI3K: Phosphatidylinositol-3 kinase
pMCAO: Permanent middle cerebral artery occlusion
PPARγ: Peroxisome proliferator-activated receptor
γ PPD: Protopanaxadiol
PPT: Protopanaxatriol
PS1: Presenilin-1
sAPPα: Secreted amyloid precursor protein-α SNP25 Synaptosomal-associated protein 25
SOD: Superoxide dismutase
SOX-2: SRY (sex determining region Y)-box 2
SYN2: Synapsin II
SOD: superoxide dismutase
TNF-α: Tumor necrosis factor-α
TrkB: Tropomyosin receptor kinase B
α7 nAChR: Alpha-7 nicotinic receptor
